# Central nervous system tumours profile at a referral center in the Brazilian Amazon region, 1997–2014

**DOI:** 10.1371/journal.pone.0174439

**Published:** 2017-04-03

**Authors:** Luis Eduardo Werneck de Carvalho, Jonathan Souza Sarraf, Aluízio Augusto Pereira Semblano, Matheus Acácio Moreira, Manuela Nascimento de Lemos, Vanessa Jóia de Mello, Moisés Hamoy, Mario Hermes Nazareth Junior, Fernando Mendes Paschoal Junior, Fernando Adami

**Affiliations:** 1 Oncológica Brasil—Ensino e Pesquisa, Belém, Pará, Brazil; 2 Laboratory of Epidemiology and Data Analysis, Faculdade de Medicina do ABC, Santo André, São Paulo, Brazil; 3 Post Gradatuation of Genetic and Molecular Biology, Universidade Federal do Pará, Belém, Pará, Brazil; 4 Centro Universitário do Estado do Pará, Belém, Pará, Brazil; 5 Laboratory of Pharmacology and Toxicology of Natural Products, Universidade Federal do Pará, Belém, Pará, Brazil; 6 Neurosurgery Department, Hospital Ophir Loyola, Belém,Pará, Brazil; Universita degli Studi di Napoli Federico II, ITALY

## Abstract

Tumours of the Central Nervous System (CNS) are an important cause of mortality from cancer. Epidemiological data on neoplams affecting the CNS are scarce in Brazil, especially in the Amazon region. The study aims at describing the histopathological profile of CNS tumours cases at a high-complexity referral cancer center. This study has described a 17-year-series profile of CNS tumours, registered at a high-complexity referral cancer center in Pará state, from January 1997 until July 2014 in the Brazilian Amazon Region. Data was gathered from histopathology reports kept in the hospital’s cancer registry and 949 cases of CNS tumours were analyzed. The most common histopathology were neuroepithelial tumours (approx. 40%) and meningioma was the most frequent especific tumor histologic subtype (22.2%). Neuroepithelial tumours were more frequent in patients with ages ranging from less than a year to 19 years, whereas metastatic tumours were prevalent in patients over 40 years of age. It was not found temporal trends during the studied period. The knowledge of these tumours profile is valuable for the understanding of cancer epidemiology in the region, since its prevalence is currently underreported and more awareness on the disease is needed.

## Introduction

Tumours of the Central Nervous System (CNS) are associated with respond high mortality rates and represent the commonest brain tumours among children and adolescents (<1–19 years of age) [1,2], but this frequency may be high in the adult population, with mean age at diagnosis 47 years [3]. Among children, 75% of such lesions are malignant whereas among adults the frequency drops to 50% [4].

Prognosis is associated with tumor site and size, age at diagnosis and tumour behavior (malignant or benign)[1,4]. Surgery is the treatment of choice for primary tumours, whenever feasible—aiming at either resecting the lesion or decompressing adjacent structures–followed by adjuvant chemotherapy and radiotherapy. However, curative treatments for malignant or for metastatic tumours are yet to be developed, with a much better rate of curability for the cases of benign tumours [1,5].

Epidemiologic data on Central Nervous System primary tumours in the Amazon region of Brazil are scarce. The Brazilian Amazon population presents strong racial miscegenation mainly between different indigenous ethnic groups, Portuguese and African descendants, which resulted not only in unique sociocultural features but, probably, also in a particular gene pool that may present distinct manifestations for cancer susceptibility and ethology [6]. Therefore, it is important a survey of these tumour profiles in the region, including the Para State.

Our study aims at describing the histopathological profile of CNS tumour cases treated at a public cancer hospital in Para State, between 1997 and 2014 and compare our findings with those of the literature.

## Material and methods

We studied 1065 central tumour registries of the Neurosurgery Service archives of the Cancer Hospital Ophir Loyola, a referral high-complexity cancer center and headquarter of the Population-Based Cancer Registry in Belem City, Para State, Brazil. Ophir Loyola Cancer Hospital—together with another public health center in the same city and the public cancer center of Santarem City—represents the main oncologic service network of our state.

Data was obtained from the histopathological reports. Those that did not provide information on location of lesion, such as whether the tumour site was the CNS or the peripheral nervous system, were excluded. The personal information of patients were anonymized through data extraction protocol that did not have nominal identification of the same, only the number of care protocol in the hospital. Data extraction and collection was performed using Microsoft Excel software. Tumours were then classified according to the WHO 2007 into neuroepithelial tissue, meninges, metastatic, cranial and paraspinal nerves, germ cells, lymphomas, hematopoietic neoplasias [7] and others.

CNS tumour frequency and classification was organized by gender (male and female), age range (<20, 20–39 and ≥ 60 years) and year of diagnosis in groups of three years. The absolute frequency of tumours was also obtained according to year of diagnosis. Statistical data analysis was performed using STATA v. 12.1 (Stata Corp.; CollegeStation, TX, USA) and graphs were made using the GraphPadPrisma v.5.0 software (GraphPad Software, Inc, San Diego, CA, USA). This study has been approved at Ophir Loyola ethic committee, number 385.928 protocol.

## Results

We excluded 78 out of the 1027 registries of central tumours due to incomplete information in the reports for determining whether they were located in the CNS or in the peripheral nervous system. Therefore, we have analyzed 949 registries of CNS tumours, representing 92.4% of the reports.

The subclassification distribution for all hystological types can be found on S1–S3 Figs. And the most frequently affected histologies were neuroepithelial tissue (~ 40%), followed by the meninges (25%), secondary CNS metastasis (12%), sellar region (10%), cranial and paraspinal nerves (7%), germ cells (1%), lymphomas and hematopoietic neoplasms (1%). The category “others” represented 5% of the tumour totals.

**Fig 1:**
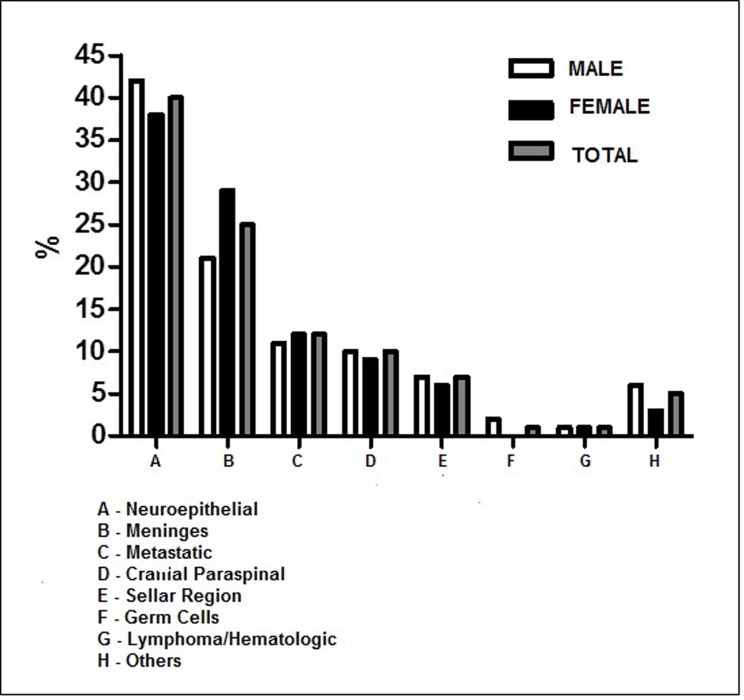
Relative frequency (%) of CNS tumor types according to gender. Hospital Ophir Loyola, 1997–2014.

Distribution according to gender was similar, except for neuroepithelial tumours which were more frequent in males (42%) than in females (38%), whereas neoplasms of the meninges were more common in females (29%) than in males (21%) **(Fig 1).** When meningiomas are removed from the meningeal tumour group the proportion of men and women changes, passing to men (4%) than women (3%).

**Fig 2:**
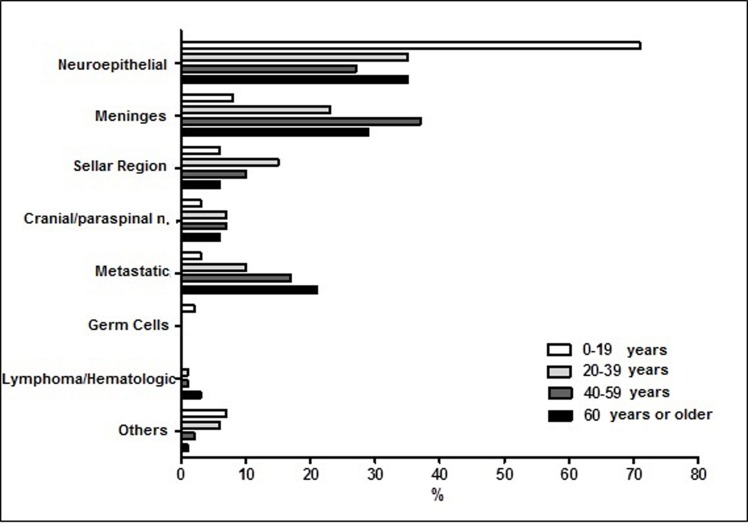
Relative Frequency (%) of CNS tumor types, according to age range. Hospital Ophir Loyola, 1997–2014.

In the young population there was a clear concentration of tumours of the neuroepithelial tissue (71% of patients younger than 20). Among patients over 40 years of age the most frequent primary lesions were found in the neuroepithelial tissue and meninges, besides secondary metastases (81% of patients between 40 and 58 years, and 85% of adults older than 60) (Fig 2).

**Fig 3:**
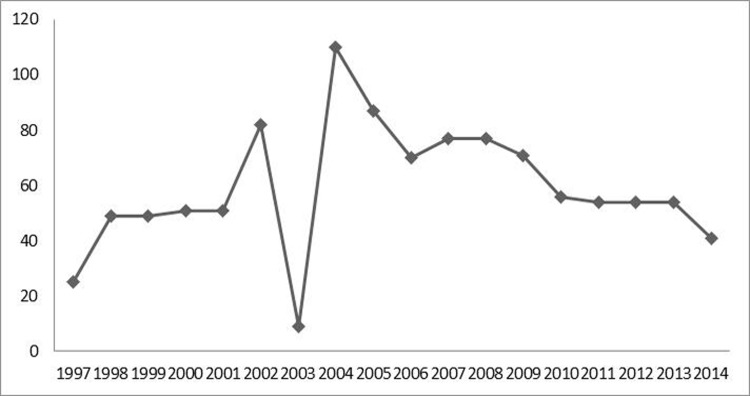
Absolute Frequency of CNS tumors per year of assessment. Hospital Ophir Loyola, 1997–2014.

**Fig 4:**
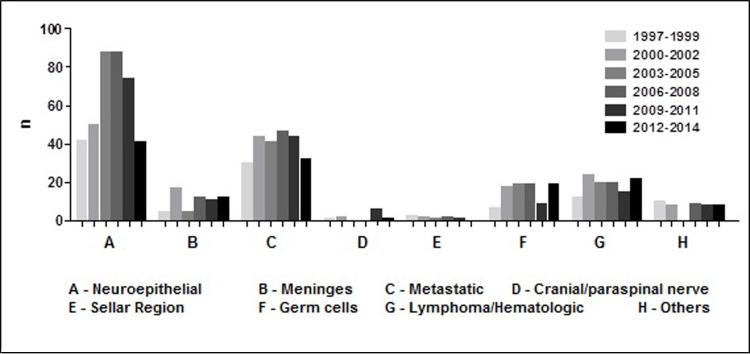
Absolute Frequency (n) of CNS tumor types per triennium of diagnosis Hospital Ophir Loyola, 1997–2014.

The absolute frequency was estimated according to year of histologic diagnosis and a divergence was observed in the year 2003, represented by only 9 cases (Fig 3). As for the analysis of diagnosis per triennium in the period comprised between 1997 and 2014, no consistent pattern emerged in the distribution of tumour cases over time (Fig 4).

## Discussion

There is no other descriptive study of CNS tumours larger than this one for the Brazilian Amazon region, as far as we know. Our results did not show significant divergences in tumour frequency between 1997 and 2014, with the predominance of neuroepithelial tumours (~40%), especially the meningioma subtype (22%), throughout the 17-year period. Furthermore, neuroepithelial tumours were more frequent in males (42%) mainly in younger ones (71% of patients younger than 20 years). Among metastic tumours the majority of them were found in older people (81% of patients between 40 and 58 years, and 85% of adults older than 60 years). Moreover, there were no time trends founded in this period of 17 years.

As for the years 2000 and 2001, we found a total of 103 new cases of CNS tumours in the Brazilian Amazon region, whereas the Population-Based Cancer Registry of the Brazilian National Cancer Institute (INCA) has only recorded 4 new cases in the region for the same period. The Cancer Registry of Para State, which is based at the Hospital Ophir Loyola, counted with 24 notifying sources of cancer cases in that period, among them, one cancer hospital, two university hospitals, nine general hospitals, eighteen labs of anatomopathology, one hematology service, and three oncology clinics [8,9]. The latter data highlights the great disparity between our findings and the number of new cases notified in 2000–2001 to the National Cancer Registry at INCA. (INCA is the coordinating authority on cancer public policies in Brazil.)

Large descriptive world series, as well as others of regional scope, have not analyzed temporal trends of CNS tumours [10,11] but one study indicated a trend of leveling off in incidence of several tumours during the study period [2]. Nevertheless, our analysis was unable to detect any temporal trend as far as CNS tumours are concerned.

Conversely, the prevalence of CNS tumour types around the world shows differential distributions among localities. For instance, in Porto Alegre city (south of Brazil), between 1995 and 2009 [10], Croatia, between 1974 and 2001 [12], and in Estonia, between 1986 and 1996 [3], Mexico between 1965 and 2014 [13], and China between 2008 and 2013[14], neuroepithelial tumors were more frequent, representing 40%, 58.3%, 52.9%, 33% and 31%respectively, of all diagnosed CNS neoplasms. In contrast, Korea in 2005 and Georgia in 2009 have shown a predominance of meningeal tumours (30% and 29.81% respectively) among diagnosed CNS tumours. In the present study we have found a prevalence of neuroepithelial tumours (~40%), which contrasts with the distribution of CNS tumour types elsewhere [10,12,15,16]

Among the tumour subtypes (WHO classification), the meningiomas clearly showed predominance over the other histologic types, in the literature [16–18]. However, the most frequent tumours are glioblastomas [3,10] and astrocitomas [10]. In our study there was a predominance of meningiomas, representing 22.2% of the cases, which agrees with the majority of the published studies [16–18].

Gender-related assessment of CNS tumours incidence did not show significant differences among males and females in the literature [10,18]. However, meningiomas are more frequent among women due to hormonal factors [18]. Our findings have shown that 29% of CNS tumours in female patients were in fact tumours of the meninges.

Neuroepithelial tumours are more frequent in patients younger than 20 years [10,16,17]. In the study, this data was confirmed in our cancer registries wherein 71% of the young patients were diagnosed with neuroepithelial tumours. As for CNS secondary metastases, our findings have also corroborated the literature by showing a prevalence of these tumours in patients over 40 years. Therefore, factors such as the incidence of several tumours and their progression rates are directly related to age and to the increase of life expectancy of the last decades [10,11,19].

During our study we were confronted by some limitations such as incomplete histopathological reports, which can lead to minor classification inconsistencies and missing about specifications and absence of a standard procedure for issuing the reports of other exams. Furthermore, in 2003 there were recorded only nine cases of CNS tumours, what sharply contrasts with the total number of cases found in any other year in our cancer registries [16]. Notwithstanding the latter, those problems did not compromise the temporal assessment of the triennium 2003–2005. Aiming at minimizing the divergences found in the reports, we have adopted the WHO classification for CNS tumours. Moreover, for example, in some our associations the predominance of females in the meninges Tumours there may be problems as the Tumours spread, since there is a clear predominance of meningiomas in this category. Thereby, it cannot be Said that for Tumours of meninges the prevalence for women is valid.

Our findings contribute to the dissemination of the epidemiological profile of a little-known population, from a singular region. We hope that our data may be useful in triggering new studies on CNS tumours in the Amazon region as well as to inform health planners on public policies for the region as far as prevention, early detection and the improvement of cancer care services are concerned. Moreover, we hope our results may be used to alert hospitals to the importance of a better control of CNS cancer registries as well as more accurate and complete clinical files.

## Conclusion

In conclusion, we may confirm that our study has not found significant differences between the prevalence and profile of CNS tumours in the Brazilian Amazon, despite its ethnic and environmental singularity, between 1997 and 2014—in comparison to other regions described in the literature. The knowledge of such profile, nevertheless, is valuable for the understanding of cancer in the region, since its prevalence is currently underreported and more local awareness on the disease is needed.

## Supporting information

S1 Fig Subclassification distribution for tumours of neuroepithelial tissue hystological types. (TIF)

S2 Fig Subclassification distribution for tumours of the meninges hystological types. (TIF)

S3 Fig Subclassification distribution for all others hystological types. (TIF)

S1 Dataset (XLS)
